# Integrated statistical and pathway approach to next-generation sequencing analysis: a family-based study of hypertension

**DOI:** 10.1186/1753-6561-8-S1-S104

**Published:** 2014-06-17

**Authors:** Jeremy S Edwards, Susan R Atlas, Susan M Wilson, Candice F Cooper, Li Luo, Christine A Stidley

**Affiliations:** 1 Molecular Genetics and Microbiology, and Chemical and Nuclear Engineering, 1 University of New Mexico, University of New Mexico Cancer Center, Albuquerque, NM 87131, USA; 2 Physics and Astronomy, Center for Advanced Research Computing, 1 University of New Mexico, University of New Mexico Cancer Center, Albuquerque, NM 87131, USA; 3 Center for Advanced Research Computing, University of New Mexico Cancer Center, 1 University of New Mexico, Albuquerque, NM 87131, USA; 4 University of New Mexico Cancer Center, Internal Medicine, 1 University of New Mexico, University of New Mexico Health Sciences Center, Albuquerque, NM 87131, USA

## Abstract

Genome wide association studies (GWAS) have been used to search for associations between genetic variants and a phenotypic trait of interest. New technologies, such as next-generation sequencing, hold the potential to revolutionize GWAS. However, millions of polymorphisms are identified with next-generation sequencing technology. Consequently, researchers must be careful when performing such a large number of statistical tests, and corrections are typically made to account for multiple testing. Additionally, for typical GWAS, the *p *value cutoff is set quite low (approximately <10^−8^). As a result of this *p *value stringency, it is likely that there are many true associations that do not meet this threshold. To account for this we have incorporated a priori biological knowledge to help identify true associations that may not have reached statistical significance. We propose the application of a pipelined series of statistical and bioinformatic methods, to enable the assessment of the association of genetic polymorphisms with a disease phenotype--here, hypertension--as well as the identification of statistically significant pathways of genes that may play a role in the disease process.

## Background

Genome wide association studies (GWAS) can be used to find associations between genetic variants and a phenotypic trait of interest. New technologies, such as next-generation sequencing, are promising to have a significant impact on our ability to find disease associations through GWAS. However, next-generation sequencing technology currently is capable of identifying millions of polymorphisms in an individual genome. Therefore, when searching for an association between a genetic polymorphism and phenotypic trait, many statistical tests are performed. Researchers must be careful when performing such a large number of statistical tests, and corrections are typically made to account for the multiple testing. Additionally, for typical GWAS the *p *value cutoff is set quite low (approximately <10^−8^). As a result of this *p *value stringency, it is likely that there are many true associations that do not meet this threshold. To account for this, newer studies have incorporated a priori biological knowledge to identify true associations that may not have reached statistical significance in light of the adjustment for the many statistical tests.

The work described herein is based on the Genetic Analysis Workshop 18 (GAW18). The GAW18 study is a family-based study drawn from 2 cohorts participating in the Type 2 Diabetes Genetic Exploration by Next-Generation Sequencing in Ethnic Samples Consortium, the San Antonio Family Heart Study, and the San Antonio Diabetes/Gallbladder Study [1]. Participants with eligible phenotype and genotype information came from 20 pedigrees. Individuals were enrolled from 1992 to 2003 and provided blood pressure, age, smoking status, and blood pressure medication status at 1 to 4 visits over the study time. The goal of our study was to identify genetic associations with hypertension phenotypes based on the data provided on the GAW18 organizing committee.

The hypertension phenotype has been investigated with GWAS in the past [2-5], and it has long been believed that hypertension is at least partially controlled through a genetic component [6]. However, the genetic association with hypertension is likely very complex, with the potential for multiple competing effects and pathways, involving renal salt processing, vascular constriction, etc [7]. The first set of studies identified a number of interesting loci [2,3]. In the Welcome Trust GWAS of hypertension, 2000 cases and 3000 controls were analyzed, and in the Framingham Heart Study 1327 individuals had blood pressure measurements performed longitudinally. No obvious associations were identified, but a number of single-nucleotide variants (SNVs) of interest were defined. A subsequent directed analysis was able to confirm an association between a single SNV and hypertension (rs1937506). Interestingly, this SNV has opposite effects in Americans of European origin versus Americans of Hispanic origin [8]. Additionally, more recent GWAS of hypertension in European and Amish populations have identified SNVs that are statistically significantly associated with hypertension [9,10].

In this study, we propose the use of a pipelined series of methods to enable the assessment of the association of genetic polymorphisms with hypertension, as well as to identify the pathways of genes that may play a role in this disease. To perform our analysis, we first removed polymorphisms from the analysis that were not within or near (± 1 kilobase [kb]) exons. Then we performed a family-based association analysis between the genotypes and 5 hypertension-related phenotypes while correcting for potential confounding factors (ie, age, sex, smoking, hypertension medication). Lists of genes that were most likely related to hypertension were identified and pathway analysis was performed on these gene lists.

## Methods

### Cohort

The GAW18 study is a family-based study drawn from 2 cohorts participating in the Type 2 Diabetes Genetic Exploration by Next-Generation Sequencing in Ethnic Samples Consortium, the San Antonio Family Heart Study, and the San Antonio Diabetes/Gallbladder Study [1]. Details of the GAW18 data set are available elsewhere (see http://www.gaworkshop.org/gaw18/index.html).

### Phenotypes and covariates

At each visit systolic blood pressure (SBP) and diastolic blood pressure (DBP) were measured, and information on use of blood pressure medication, smoking status, and age were also collected. We defined 5 phenotypes to allow multiple avenues for assessing genetic components involved with blood pressure: hypertension status, SBP, DBP, average yearly change in SBP (SBP slope), and average yearly change in DBP (DBP slope).

Hypertension was defined as SBP >140 mm Hg, DBP >90 mm Hg, or the individual reported taking blood pressure medication. Because individuals had from 1 to 4 visits, the basic hypertension variable was obtained as ever versus never hypertensive across all visits. Covariates were obtained from the same visit, with the first eligible visit used when multiple visits were eligible. For individuals who were never identified as hypertensive, the covariates from the final visit were used.

SBP and DBP values were available at most visits. If an individual indicated taking blood pressure medication, then a standard adjustment of 10 mm Hg and 5 mm Hg for SBP and DBP, respectively, was added [11]. For analyses with a single blood pressure variable, the SBP and DBP values from an individual's first visit were used. To assess blood pressure changes over time, the slope from a linear regression of blood pressure as a function of time was obtained for SBP and DBP for each participant, yielding a simple summary measure of average change per year for each individual.

### Genotypes

We used the next-generation sequencing genotyping data from the 483 Hispanic individuals provided by the Genetic Analysis Workshop organizers and the imputed genotypes from 961 individuals in 20 large pedigrees. We used the genotype calls provided by the organizers for the 959 individuals, which included more than 8 million polymorphisms on the odd-numbered chromosomes. This original set was filtered down to include only polymorphisms inside of exons or within 1 kb of an exon. Additionally, because we performed a pathway analysis with the genes that contained polymorphisms statistically most likely to be associated with hypertension, only polymorphisms associated with a gene were applicable to our analysis. Polymorphisms outside of genes were excluded from the statistical analysis. We identified polymorphisms that were within or near exons based on the UCSC RefSeq annotation GRCh_37. The filtering identified 849,517 polymorphisms for analysis. Standard screening for Hardy-Weinberg equilibrium was not used, as both the family-based nature of this study and admixed populations, such as this Hispanic population, may violate Hardy-Weinberg equilibrium as a result of the admixture process [12].

### Statistical methods for the association analysis

The primary purpose of the association analysis was to identify potentially important genes to inform the subsequent pathway analysis. Thus, we used 5 phenotypes to allow the assessment of different pathways for the development of hypertension and other features concerning blood pressure. The individual SNVs were assessed for association with the various phenotypes. All standard genetic models were assessed, including additive, dominant, recessive, and heterozygote advantage. Logistic regression was used to assess the binary traits, whereas linear regression was used for the quantitative traits (SBP, DBP, SBP slope, and DBP slope). Standard adjustment variables, age, age^2^, and gender, were included, along with smoking status [13]. All analyses took into account family structure and used the enhanced extended pedigree analysis in Golden Helix (Bozeman, MT). Standard Bonferroni adjustment for multiple comparisons was not used, but because the primary purpose of the association analysis is to inform a pathway analysis, the ranking of variables on the basis of *p *values is not affected. Data summaries, such as slopes from linear regression, and covariate values were obtained utilizing SAS version 9.3.

### Pathway analysis methodology

Pathway analyses were performed using Ingenuity Pathway Analysis (IPA) pathway analysis software (Ingenuity Systems, Inc., Redwood City, CA, http://www.ingenuity.com). Identified pathways were ranked in order of increasing *p *value. The unadjusted pathway *p *values were computed using the hypergeometric distribution (probabilities computed without replacement), using a right-tailed Fisher's exact test to assess the significance of a given pathway in relation to the input data set. The *p *value computation takes into account the number of input molecules, the size of the requested pathway, and the total number of molecules from the IPA knowledge base that could potentially be included in the pathway. Connections between molecules in the reported pathways reflect experimentally validated evidence of pairwise interactions from the literature, as annotated in the IPA knowledge base. Pathway *p *values (reported as score = −log_10_[*p *value]) correspond to a level of confidence that a given pathway and its molecular interactions did not arise by chance, but instead correspond to biologically meaningful relationships among the input genes associated with the given phenotype. For example, a pathway with a *p *value of 10^−9 ^would have a 1 in 1 billion chance of arising purely by chance, if the input molecules had been chosen randomly from the underlying knowledge base.

For each set of SNVs significantly associated with a phenotype, we derived the corresponding gene lists for input to the IPA software by mapping SNVs to the nearest gene. A series of cutoffs were applied in order to explore the effects of varying numbers of genes and *p *value thresholds. Each cutoff resulted in a separate gene list and distinct pathway analyses.

Pathway analyses were performed so as to construct the most parsimonious pathway for each set of genes, up to a maximum of M_IPA _interacting molecules (proteins, chemicals), and such that the maximum number of genes from the input set is included in a given pathway. In the results reported here, M_IPA _was chosen to be either 70 or 140.

## Results

**Figure 1:**
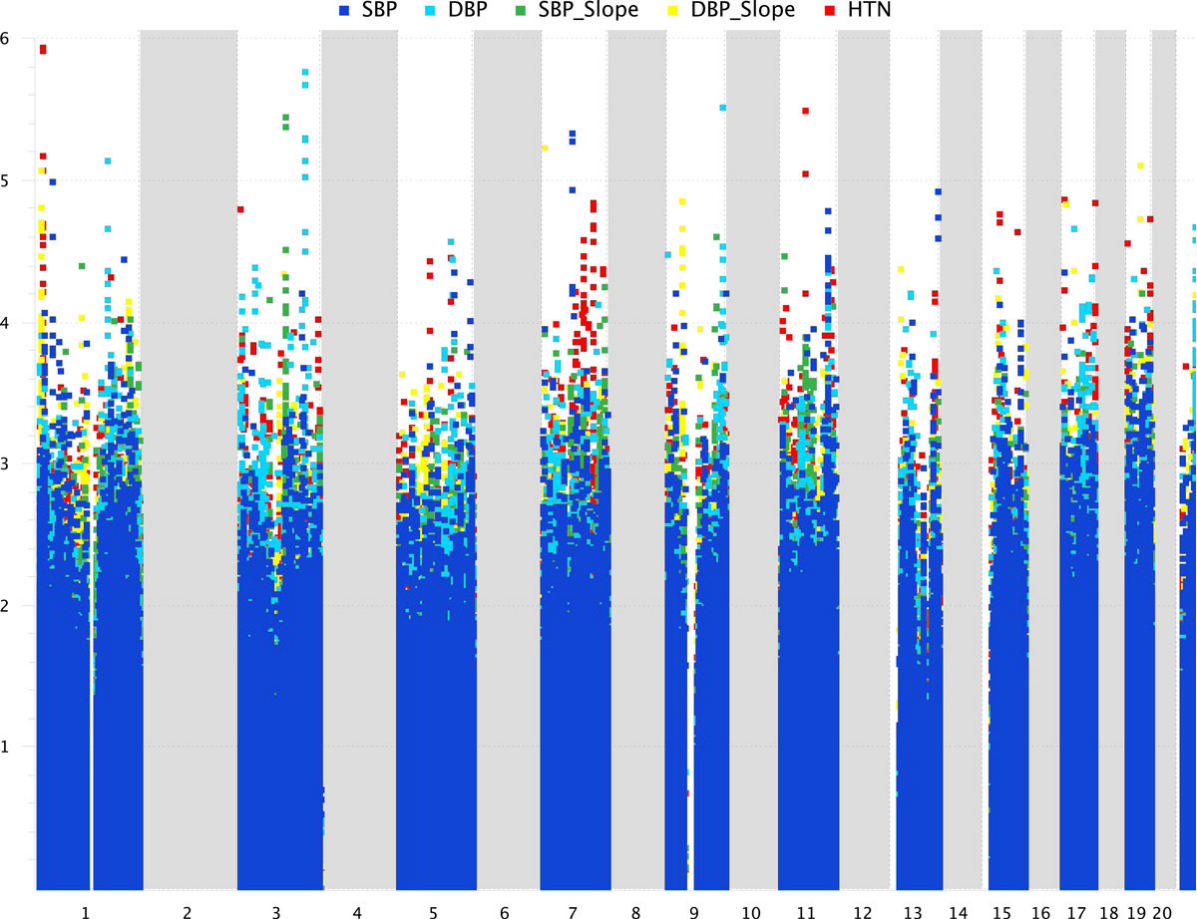
**Genome wide association scans for 5 different phenotypes related to hypertension**. For each of the 5 phenotypes, the -log_10 _of the *p *value associated with each SNV in (or near) the coding regions. Data from only odd-number chromosomes was provided as part of the GAW18 project; consequently, there is no information for any of the even-number chromosomes, which appear as blank regions on the plots. *HTN*, hypertension.

Although the median age of participants at enrollment was only 38 years, 40% were identified as hypertensive (Table 1). Participants were more likely to be female (57.9%) and 21.7% were current smokers at the time of enrollment. The cross-sectional analysis included 841 people, with 634 with multiple visits and thus eligible for the analysis of change in blood pressure. Average annual change in SBP and DBP were 0.95 and 0.23 mm Hg, respectively. Below we will first present results from the family-based logistic regression and linear regression models for the 5 phenotypes identified SNVs and, thus, genes that appeared to be associated with the phenotypes (Figure 1). We will then apply a pathway analysis to these lists of genes to identify functional connections between the most significantly associated genes.

**Table 1:** Summary information on demographic and phenotypic variables

Demographic or phenotypic variable	Summary measures
*Information from first visit (n = 841)*	
Sex (% female)	57.9
Age (median [min, max]) in years	38 (16, 94)
Smoking status (% current smoker)	21.7
Taking blood pressure medication (%)	10.0
*Phenotypes: cross-sectional (n = 841)*	
Ever hypertensive (%)	40.0
SBP (median [25^th^, 75^th ^percentiles]) (mm Hg)	118 (110, 130)
DBP (median [25^th^, 75^th ^percentiles]) (mm Hg)	72 (65, 78)
*Phenotypes: average yearly change (n = 634)*	
SBP (mean [std]) (mm Hg)	0.95 (2.09)
DBP (mean [std]) (mm Hg)	0.23 (1.27)

### SBP

We identified ten SNVs within genes with -log_10_(*p *value) >4.5. Two of these SNVs were on chromosome 1 (1p34/CLSPN), 3 on chromosome 7 (7q11/AUTS2), 2 on chromosome 11 (11q23/HTR3B, 11q23/TMPRSS5), and 3 on chromosome 13 (13q34/LAMP1). Interestingly, 2 of these proteins are on the cell membrane (HTR3B, TMPRSS5) and 1 is identified as an ion channel (HTR3B).

### DBP

Six genomic regions were associated (-log_10_[*p *values] >4.5) with the DBP quantitative trait. These regions are on chromosomes 1q, 3q, 5q, 9q, 17q, and 21q. The chromosome 1 region is 1q24 and the SNVs are in the *C1orf114 *and *SLC19A2 *genes. The *SLC19A2 *gene encodes a protein that is annotated as a transporter and it exists on the cell membrane. The chromosome 3 region with SNVs of potential significance is 3q25 and the SNVs are within 3 adjacent genes (*SMC4, IFT80*, and *KPNA4*). The chromosome 5 SNV is in *FBN2 *which lies on 5q23. There are 2 chromosome 9 SNVs, both of which are in *PRDM2 *on 9q34. A single SNV of potential significance exists on chromosome 17 (17q11) and is in the *NUFIP2 *gene. And finally, the 3 SNVs on chromosome 21 (21q22) are in the *TRPM2 *gene, which is a transient receptor potential cation channel that is involved in Ca^++ ^and Na^+ ^transport. It should be noted that 6 of the top 7 SNVs (with functional annotations) are transporters or ion channels.

### SBP slope

From our family-based analysis, we identified 2 genomic regions associated (-log_10_[*p *values] >4.5) with the SBP slope quantitative phenotype, 3q13 and 9q32. The 3 SNVs on the chromosome 3 region were in the *WDR52 *gene, and 2 SNVs on 9q32 were in the *SNX30 *gene. One of the most significant SNVs associated with a change in SBP and of particular interest is VAV3 (*p *value = 3.97 × 10^−5^). This SNV was also identified as a key gene in association with a change in DBP and it has been implicated in hypertension and tachycardia in a mouse model [14].

### DBP slope

Five genomic regions were associated (-log_10_[*p *values] >4.5) with the DBP slope quantitative phenotype, 1p36, 7p22, 9p21, 17p13, and 19q13. There are 6 SNVs on chromosome 1 in the *KIF1B *gene. The chromosome 7 SNV is in the *SDK1 *gene. On chromosome 9 there are 13 SNVs in 3 adjacent genes that are potentially associated with the phenotype. These SNVs are in the *SMU1, DNAJA1*, and *APTX *genes. On chromosome 1 there is a single SNV with potential significance within the *PER1 *gene. Finally, there is a single SNV in the *PEPD *gene on chromosome 19.

### Hypertension

We identified the most genomic regions associated (-log_10_[*p *values] >4.5) with the hypertension binary phenotype. There were 11 regions: 1p36 (PTCHD2 and C1orf187), 3p26 (IL5RA), 7q22 (GATS and STAG3), 7q31 (LMOD2 and WASL), 11q12 (OR8U1 and OR8U8), 15q15 (PLA2G4E), 15q25 (ADAMTSL3), 17p13 (PITPNM3), 17q25 (CCDC57), 19p13 (WDR18), and 19q13 (PPP6R1). Although none of these regions contains a gene that has a known biological relationship with hypertension, when the *p *value cutoff is increased, a number of genes that are associated with hypertension appear on this list. Furthermore, many of these potentially interesting genes also appear on the other phenotype lists.

### Genes associated with phenotype

The 5 phenotypes defined in this study were constructed in an attempt to capture different manifestations and etiologies of hypertension. Consequently, the genes emerging from the association analysis for each phenotype are expected to display distinct biological patterns, in principle traceable to different underlying patterns of disease and pathway dysregulation. In addition, there is the possibility of observing *commonalities *across phenotypes, global patterns that transcend specific phenotypic definitions. Several such patterns emerged from our analysis of the gene lists for the various phenotypes.

In particular, of the 5 hypertension phenotypes considered, DBP and hypertension yielded the largest numbers of significant genes (*N *= 34 and 38, respectively) with a *p *value cutoff of 10^−4^. Genes with family annotations of "ion channel" or "transporter" are notable because of the fundamental importance of ion and salt transport processes underlying hypertension pathways [15,16]. Examples of ion channel-annotated genes derived from the DBP phenotype include *TRPM2 *(cation channel), *SCN11A *(sodium ion channel), *ITPR1 *(Ca^+2^-release mediator), and, (implicated in hypertension [2,17]. *RYR3 *also appears in the 5 phenotype intersection list, as does the NALCN sodium leak channel. Cellular transporters are present within several phenotypes, and of particular note, they span 3 distinct molecular motor protein subfamilies: KIF1B (kinesin; DBP slope); DNAH14 (dynein; SBP slope), DNAH17, and DNAH9 (dynein; 5 phenotype intersection); and MYO1D (myosin; 5 phenotype intersection). MYO1D has been associated with hypertension [18], and phosphorylation of the myosin light chain, mediated by Ca^+2^, is necessary for the regulation of vascular small muscle contraction [19]. VAV3 was identified in both the DBP slope and SBP slope phenotypes as noted above.

### Pathways associated with phenotypes

**Figure 2:**
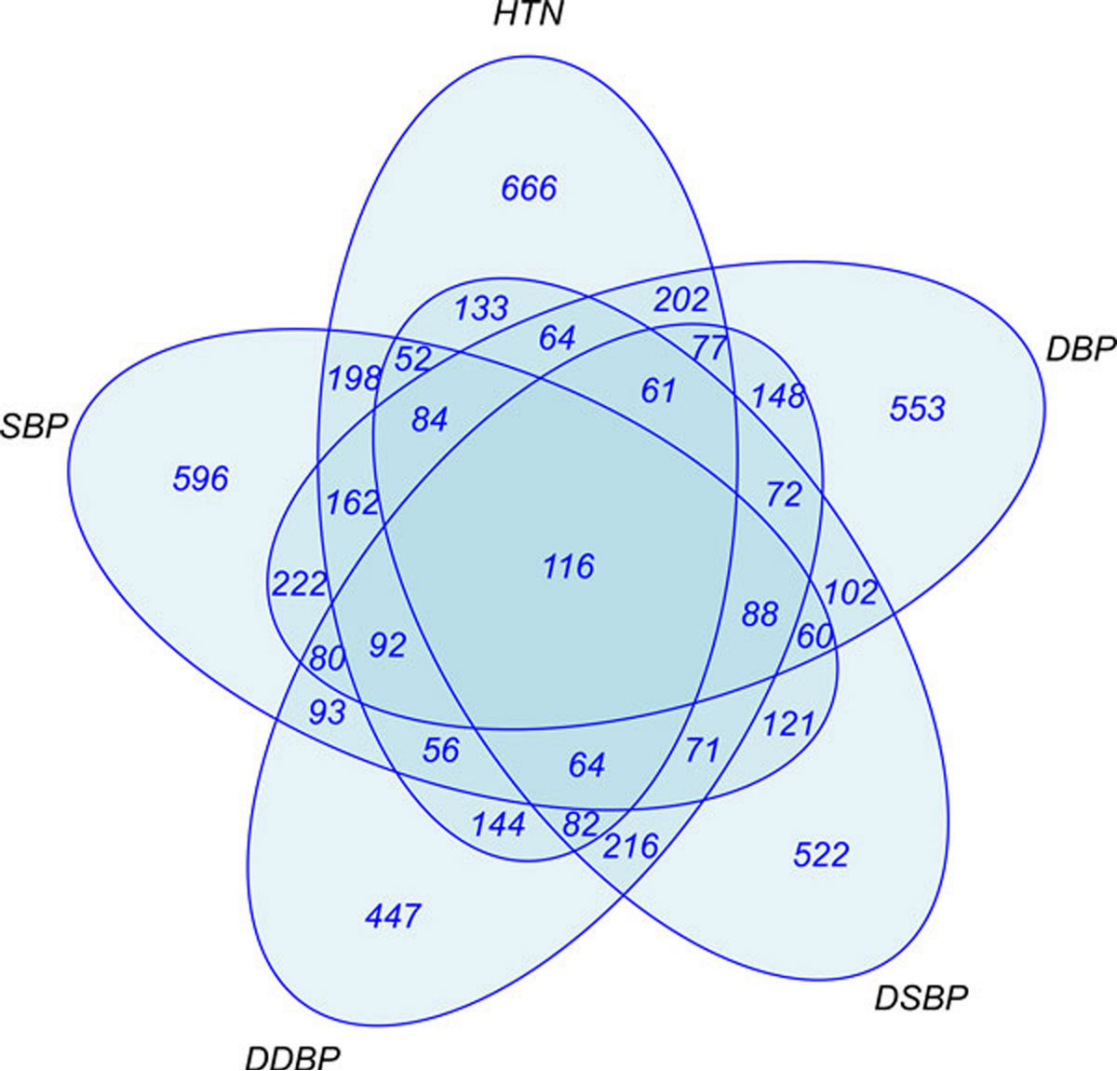
**Venn diagram of top genes for each phenotype**. A list of all SNVs that had *p *values <0.01 was analyzed. All the genes in these regions were tabulated and a Venn diagram was constructed to identify which genes existed on each of the 5 different lists. *DDBP*, change in DBP overtime; *HTN*, hypertension; *DSBP*, change in SBP overtime

**Figure 3:**
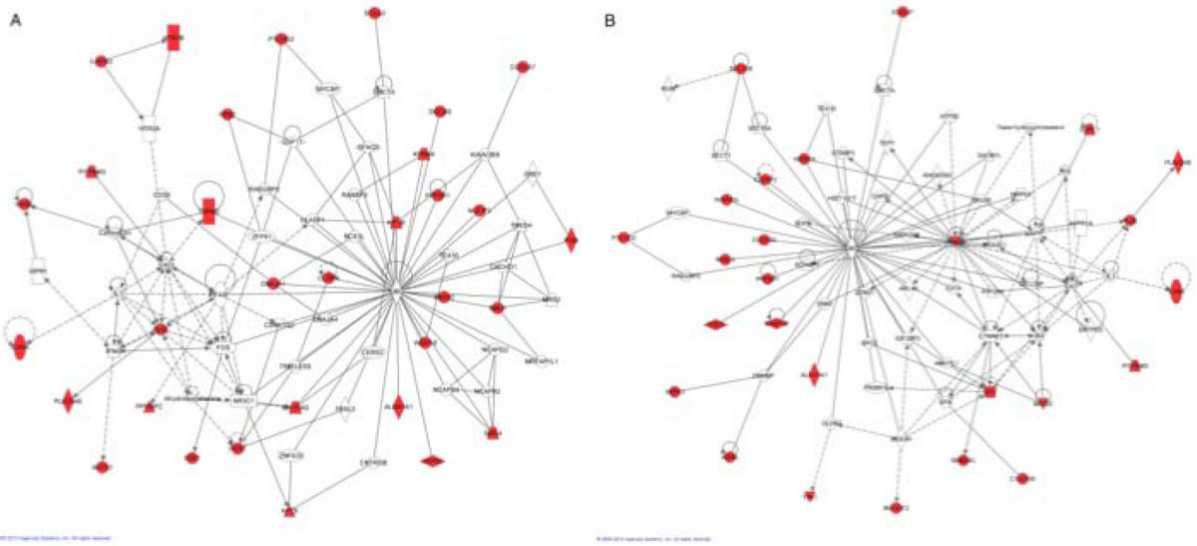
**Pathways generated by lists of genes from the association analysis**. A, Pathway analysis of top 40 statistically significant genes (*p *<3.5 × 10^−5^) derived from the union of the genes across all 5 phenotypes, M_IPA _= 70. Pathway score = 77, corresponding to *p *= 10^−77^. This was the only statistically significant pathway obtained for this set of genes. Molecules in red correspond to genes (*N *= 30, 75%) from the input list of 40. Functional annotations for this pathway include *cell death and survival, gastrointestinal disease*, and *inflammatory disease*. B, Pathway analysis of the top 38 statistically significant genes (*p *<1.0 × 10^−4^) derived from the hypertension phenotype, M_IPA _= 70. Pathway score = 62, corresponding to *p *= 10^−62^. This was the only statistically significant pathway obtained for this set of genes. Molecules in red correspond to genes (*N *= 25, 66%) from the input list of 38. Functional annotations for this pathway include *cellular assembly and organization, cellular function and maintenance*, and *cell death and survival*.

The 5-way intersection gene list reflecting genes common across all 5 phenotypes with a *p *value less than 0.01 (*N *= 116) and the most significant 40 genes (*p *<3.5 × 10^−5^) derived from the union of the genes across all 5 phenotypes were subjected to pathway analysis. We imported each phenotype gene list, as well as the top 40 list from the phenotype union, and the *N *= 116 intersection of top phenotype genes (Figure 2), into the IPA software. Figure 3 illustrates 2 of the top-scoring pathways. In these pathways, UBC (ubiquitin) and Ca^+2 ^are prominent hubs, even though these molecules were not included in the input gene lists derived from the original list of statistically significant SNVs. UBC, located on (even-numbered) chromosome 12 (12q24.3) and thus not directly accessible through data provided in this study, appears as a hub in almost all pathways, while the calcium divalent cation, required for smooth muscle contraction, appears in 2 of the statistically significant pathways. VAV3, mentioned above, is a hub in the SBP slope pathway. Interestingly UBC controls the modulation of cell surface receptors and ion channels [20], and there is emerging evidence for its importance in the initiation of atherosclerosis and the proliferation of cardiovascular disease [21,22]. Although it is intriguing that UBC appeared in nearly all of the statistically significant pathways, it is clearly premature to attribute too much significance to this finding in light of the incomplete nature of the source data set, and the wide range of cellular processes affected by ubiquitin protein modification in general.

## Conclusions

In summary, we have demonstrated--in the context of the GAW18 hypertension data set--a proposed methodology for the unified analysis of next-generation sequence data. This method used biostatistical methods for analyzing GWAS to inform a pathway analysis. Our approach integrates traditional GWAS-based statistical analysis in which variants and clusters of variants are used to define specific genetic disease markers, coupled with a broader systems biology and pathway-based approach that can potentially shed light on the diverse biological origins of complex diseases such as hypertension. It is important to note that the specific results reported here on the genetics of hypertension need to be reassessed with a more complete data set, as the GAW18 data set included only odd-numbered chromosomes, thus omitting half of the genes in the genome. Because pathway analyses utilize information for every gene, either a positive association or none, this major omission clearly impacted the analyses reported here. That is, the omission of a gene as input to a pathway analysis is effectively an assessment of its lack of importance. Because genes on even-numbered chromosomes could not be assessed in this study, and the underlying database of biological interactions is agnostic with respect to input gene lists, the pathways that were most likely to be identified were those with a preponderance of genes on odd-numbered chromosomes. Even in studies where complete information is available as input to an analysis, it is important to appreciate that the results of pathway analyses are at best a starting point for further biological studies, as they are limited by biases and incomplete data in the underlying knowledgebase. The possibility also exists that one may identify a "significant molecule" that simply reflects a pervasive underlying process, rather than one that is truly important and of functional significance in the context of a particular disease. Nevertheless, a number of suggestive results emerged from our analyses that represent a promising starting point for future studies of hypertension using expanded next-generation data sets and the integrated computational methodology described here.

## Competing interests

The authors declare that they have no competing interests.

## Authors' contributions

JSE, CAS, LL, and SRA designed the overall study. JSE and CAS conducted statistical analyses. SRA performed the pathway analysis. JSE, CAS, and SRA drafted the manuscript. SMW and CFC assisted with the analysis. All authors read and approved the final manuscript.
